# Utility of diffusion weighted imaging-based radiomics nomogram to predict pelvic lymph nodes metastasis in prostate cancer

**DOI:** 10.1186/s12880-022-00905-3

**Published:** 2022-11-04

**Authors:** Xiang Liu, Jingyi Tian, Jingyun Wu, Yaofeng Zhang, Xiangpeng Wang, Xiaodong Zhang, Xiaoying Wang

**Affiliations:** 1 Department of Radiology, Peking University First Hospital, Beijing, 100034 China; 2 Department of Radiology, Beijing Water Conservancy Hospital, Beijing, 100036 China; 3 Beijing Smart Tree Medical Technology Co. Ltd, Beijing, 100011 China

**Keywords:** Prostate cancer, Lymph nodes, Radiomics, Nomogram, Magnetic resonance imaging

## Abstract

**Background:**

Preoperative pelvic lymph node metastasis (PLNM) prediction can help clinicians determine whether to perform pelvic lymph node dissection (PLND). The purpose of this research is to explore the feasibility of diffusion-weighted imaging (DWI)-based radiomics for preoperative PLNM prediction in PCa patients at the nodal level.

**Methods:**

The preoperative MR images of 1116 pathologically confirmed lymph nodes (LNs) from 84 PCa patients were enrolled. The subjects were divided into a primary cohort (67 patients with 192 positive and 716 negative LNs) and a held-out cohort (17 patients with 43 positive and 165 negative LNs) at a 4:1 ratio. Two preoperative pelvic lymph node metastasis (PLNM) prediction models were constructed based on automatic LN segmentation with quantitative radiological LN features alone (Model 1) and combining radiological and radiomics features (Model 2) via multiple logistic regression. The visual assessments of junior (Model 3) and senior (Model 4) radiologists were compared.

**Results:**

No significant difference was found between the area under the curve (AUCs) of Models 1 and 2 (0.89 *vs.* 0.90; *P* = 0.573) in the held-out cohort. Model 2 showed the highest AUC (0.83, 95% CI 0.76, 0.89) for PLNM prediction in the LN subgroup with a short diameter ≤ 10 mm compared with Model 1 (0.78, 95% CI 0.70, 0.84), Model 3 (0.66, 95% CI 0.52, 0.77), and Model 4 (0.74, 95% CI 0.66, 0.88). The nomograms of Models 1 and 2 yielded C-index values of 0.804 and 0.910, respectively, in the held-out cohort. The C-index of the nomogram analysis (0.91) and decision curve analysis (DCA) curves confirmed the clinical usefulness and benefit of Model 2.

**Conclusions:**

A DWI-based radiomics nomogram incorporating the LN radiomics signature with quantitative radiological features is promising for PLNM prediction in PCa patients, particularly for normal-sized LNM.

**Supplementary Information:**

The online version contains supplementary material available at 10.1186/s12880-022-00905-3.

## Background

Extended pelvic lymph node dissection (ePLND) or PLND is recommended in intermediate- and high-risk prostate cancer (PCa) patients when the estimated risk for positive lymph nodes (LNs) exceeds 5% according to the European Association of Urology (EAU) guidelines [1]. To date, PLND represents the most accurate staging procedure to assess the presence of pelvic lymph node metastasis (PLNM) in PCa patients [2]. However, PLND may be associated with a higher risk of complications, and PLND at radical prostatectomy (RP) is currently performed blindly, without knowledge of the presence of metastases [3, 4].

Studies have shown that the incidence of LN involvement in patients with PCa is relatively low in patients who have undergone PLND at RP. Yaxley et al. reported that the positive rate of PLNM was only 5.5% in a series of 1,180 patients treated with ePLND [5]. Another cohort of 19,633 patients who had undergone PLND reported 505 positive LNs, translating to 2.5% of this cohort having node metastases [6]. These findings indicate that many patients are overtreated and must bear substantial adverse effects, morbidity, and health care costs. Therefore, accurate detection and identification of the LN status noninvasively and preoperatively are essential and helpful for clinicians to determine whether to perform a PLND, as well as which postoperative adjuvant therapy to use.

Pelvic multiparametric magnetic resonance imaging (mpMRI) has been accepted as the first choice for prebiopsy imaging of PCa patients, enabling the synchronous diagnosis of local nodal staging (N0/N1) [7, 8]. Diffusion-weighted imaging (DWI) is widely used to differentiate metastatic LNs from nonmetastatic LNs in patients with PCa, and the apparent diffusion coefficient (ADC) value is a critical parameter to identify the presence or absence of PLNM [9, 10]. However, their limitations are also clear. The traditional size criterion for metastatic LN evaluation on DWI images is a short diameter greater than 8–10 mm, which is considered unsatisfactory because of hyperplastic and micrometastatic LNs [11]. The suboptimal accuracy of DWI and ADC in diagnosing nodal metastasis for PCa presents a crucial challenge and opportunity to develop more accurate diagnostic methods.

Radiomics is a rapidly evolving field of research concerned with the extraction of a set of quantitative imaging features that can predict nodule and tumor behavior noninvasively, thus potentially overcoming some human limitations in diagnostic accuracy [12, 13]. However, to date, the specific PLNM prediction using radiomics analysis based on MRI remains limited.

Additionally, traditional radiomics models require radiologists to manually draw the volumes of interest (VOI), a time-consuming and challenging process [14]. Advances in deep learning techniques have facilitated the development of processes for automated and accurate lesion segmentation on MRI images and that can be used as the input to develop a radiomics model [15, 16].

Therefore, in this study, we aimed (1) to establish and validate an LN radiomics model based on automatically segmented VOIs of pelvic LNs on DWI images for preoperative PLNM prediction and (2) to further explore the clinical value of the radiomics model compared with quantitative radiological features and radiologists.

## Materials and methods

This retrospective study was approved by our institutional review board, and the requirement for informed consent was waived (2021-060).

### Study sample

A total of 537 consecutive patients with pathologically confirmed PCa between January 2017 and June 2021 were identified for this study. The patient inclusion criteria were as follows: (1) patients who had undergone ePLND/PLND at RP; (2) patients with at least one pathologically confirmed PLNM; (3) patients without preoperative treatment and other coexisting malignancies; (4) patients who had undergone MRI examinations including DWI performed less than 30 days before RP. Patients were excluded for the following reasons: (1) patients without available clinical and pathological characteristics; (2) patients with poor image quality (images with motion or susceptibility artifacts).

**Fig. 1:**
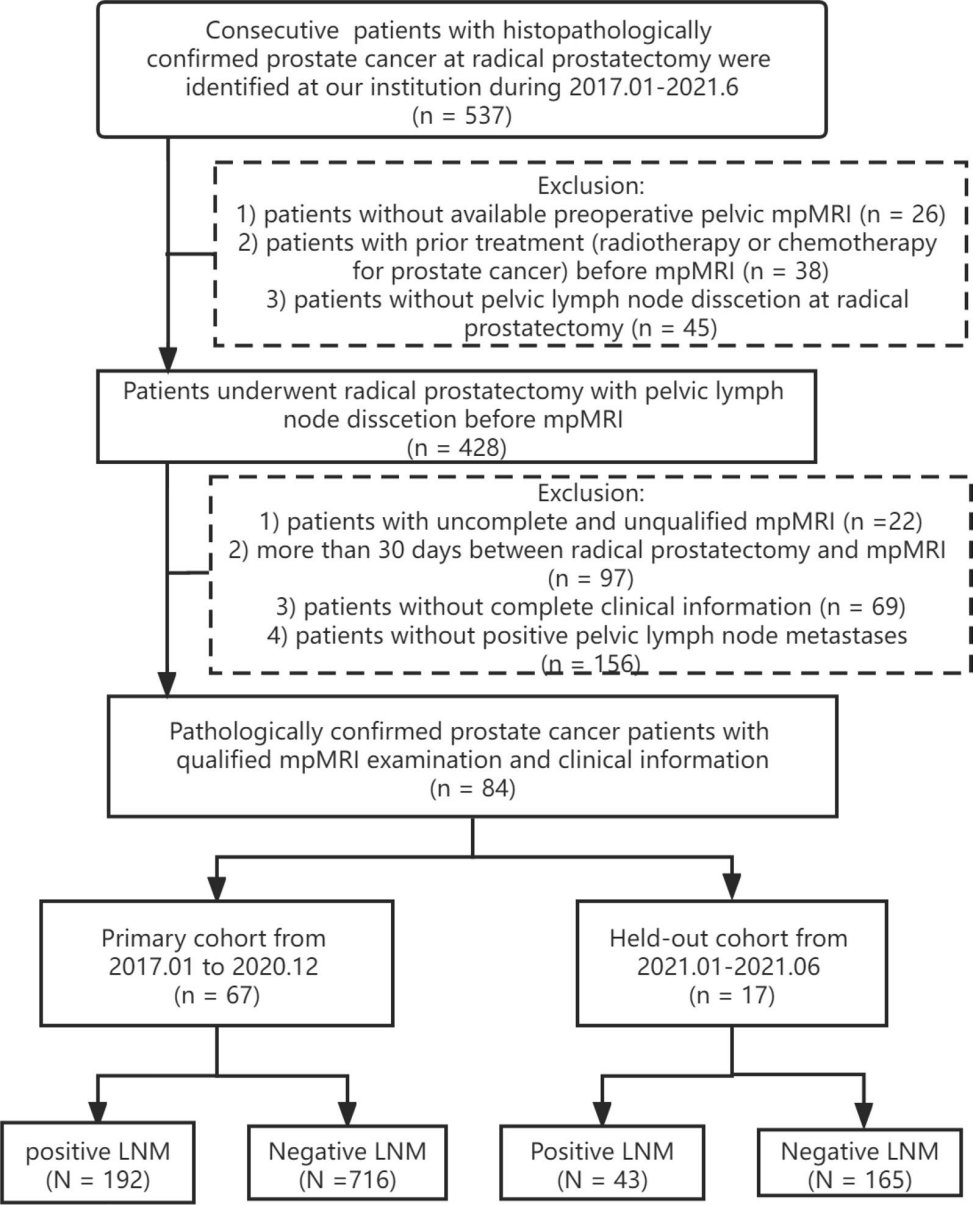
The flow chart of patient enrollment. LNM: lymph node metastases. The clinicopathologic characteristics of the patients were obtained from the medical records, including age, the Gleason score, prostate-specific antigen (PSA) level, and clinical and pathological T stage. The preoperative radiological features of the pelvic LNs include the ADC value, LN size (the shortest and longest diameters), and LN volume

The preoperative MR images of 1116 pathologically confirmed LNs from 84 PCa patients were finally enrolled (Fig. 1). We divided the subjects into a primary cohort (January 2017 to December 2020) and a held-out cohort (January 2021 to June 2021) at a ratio of 4:1 according to the MRI examination time. The primary cohort included 67 patients [median age (interquartile range): 68 (62, 74) years] with 908 pelvic LNs (positive LNM, n = 192; negative LNM, n = 716), and the held-out cohort included 17 patients [70 (65, 72) years] with 208 LNs (positive LNM, n = 43; negative LNM, n = 165).

The clinicopathologic characteristics of the patients were obtained from the medical records, including age, the Gleason score, prostate-specific antigen (PSA) level, and clinical and pathological T stage. The preoperative radiological features of the pelvic LNs include the ADC value, LN size (the shortest and longest diameters), and LN volume. All the enrolled patients were anonymous.

### MRI acquisition

To eliminate contents from the bowel, the patients were prepared to self-administer a cleansing enema (Folium Sennae) the day before their scheduled mpMRI. All the patients had undergone pretreatment mpMRI using one of two 3.0 T scanners (Achieva, Philips Health care, The Netherlands; Discovery HD 750, GE Healthcare, USA) and a 16-channel matrix torso coil. The standard mpMRI protocol at our institution included transverse T1-weighted images (T1WI), transverse T2-weighted images with fat suppression (fT2WI), dynamic contrast-enhanced (DCE) images and axial DWI with the reconstruction of ADC maps. DWI images with two b values (800 s/mm^2^ and 0 s/mm^2^) were obtained, and the ADC parameters were calculated and constructed based on the two b values. Detailed information on the DWI parameters is presented in Table 1.

**Table 1:** The detailed imaging parameters for diffusion-weighted imaging

Parameters	3.0 T discovery	3.0 T achieva
B value (s/mm^2^)	800	800
Imaging matrix	256 × 256	156 × 180
Echo time (ms)	60	54
Repetition time (ms)	4000	3300
Field of view (mm^2^)	450 × 366	512 × 356
Section thickness (mm)	8	7
Number of slices	25	24

### Pelvic LN segmentation

Once an LN is visualized in the setting of a patient with PCa, many potentially useful features can be used to determine whether it is involved with metastases [17]. Therefore, we used a previously trained 3D U-Net segmentation model based on deep learning to automatically segment the visible pelvic LNs on DWI images[18]. The segmented LNs located in the presacral, common iliac, internal iliac, external iliac, and obturator fossa sites were selected and used for PLNM prediction. The quantitative radiological features, including the ADC value, volume, short diameter, long diameter, and short-to-long diameter ratio of the segmented pelvic LNs based on deep learning, were measured automatically.

### Standard reference

The standard reference of the status of pelvic LN was a node-by-node correspondence between DWI and the final pathological assessment of ePLND/PLND. If not all the LNs were metastatic or nonmetastatic at one site, the location of the metastatic LN was discussed by one dedicated uropathologist and an expert radiologist. Any LNs without a confirmed pathological status were excluded.

### Radiomics analysis

The radiomics analysis workflow included the following four steps: (1) feature extraction, (2) feature selection, (3) cross-validation and 4) predictive performance evaluation.

The radiomics features were extracted using the PyRadiomics package in Python. In total, 1070 radiomics features were extracted from each VOI, containing 840 texture features, 216 first-order statistical features, and 14 shape-based features (Additional file 1: S1 and Table S1).

The radiomics models were developed for the primary cohort according to the following 5 steps: (i) data balance (3 methods); (ii) data normalization (4 methods); (iii) dimension reduction (2 methods); (iv) feature selection (3 methods, each with the top 1–20 features); (v) classification (6 methods) (Additional file 1: Table S2). Finally, 8640 (3 × 4 × 2 × 3 × 20 × 6) models were built. Fivefold cross-validation was applied to the primary cohort for model training and validation, and the held-out cohort was used to investigate the predictive power of the radiomics models in metastasis prediction for each LN. All the work related to radiomics model building and testing was completed using Feature Explorer Pro (FAEPro, v0.3.4) in Python (v3.6.0) [19].

### Development of PLNM prediction models

#### Model 1: quantitative radiological features of the LNs on DWI images

The difference in radiological features between PLNM and non-PLNM was first assessed by univariate analyses. Next, features with *P* < 0.05 in the univariate analyses were entered into multivariate analyses to build Model 1 using forward selection logistic regression for PLNM prediction.

#### Model 2: radiomics and radiological features based on DWI images

The radiomics model achieving the best performance among the 8640 built models in the fivefold cross-validation was finally selected as the best model for PLNM prediction. A combined radiomics model (Model 2) was developed based on the incorporation of radiomics features and quantitative radiological features using multivariable logistic regression analysis in the primary cohort.

#### Models 3 and 4: visual assessment of the radiologists

Two junior radiologists (with 5 and 6 years of reading experience, respectively) and two senior radiologists (with 10 and 15 years of reading experience, respectively) participated in reading the mpMR images. The consensus reached by the two junior radiologists to determine the metastatic status of LNs was recorded as the result of Model 3. Similarly, the consensus reached by the two senior radiologists was regarded as the result of Model 4. All the available imaging and clinical information were unblinded to the four radiologists.

### Evaluation of the PLNM prediction models

The diagnostic performance of the models in the held-out cohort was divided into three parts: discrimination ability, clinical usefulness and clinical benefit. The discrimination performance was assessed using the receiver operating characteristic (ROC) curve, and the corresponding area under the curve (AUC), accuracy, sensitivity, specificity, positive likelihood ratio (PLR), and negative likelihood ratio (NLR) were also calculated. Additionally, we divided the held-out cohort into two subgroups according to the short diameter of each LN—(a) LNs with a short diameter of ≤ 10 mm and (b) LNs with a short diameter of > 10 mm—to facilitate subgroup analysis of PLNM discrimination.

To provide a visualized and individual tool to predict the probability of PLNM, nomogram analysis was conducted based on Models 1 and 2. The C-index was calculated to assess the discrimination performance of the nomograms. The calibration curve was plotted to explore the consistency between the nomogram-predicted probability of PLNM and actual results accompanied by Hosmer–Lemeshow tests. Decision curve analysis (DCA) was adopted to assess the clinical benefits of the four models at a range of threshold probabilities [20].

### Statistical analysis

After testing normality, the Mann–Whitney U test was used to assess the characteristic differences between patients in the primary and held-out cohorts (age, F-PSA, T-PSA, F/T PSA) and between LNs in the LNM and non-LNM groups (ADC value, LN volume, short diameter, long diameter, and short-to-long diameter ratio) using Statistical Package for Social Sciences, SPSS 19.0 (SPSS Inc., Chicago, IL, USA). ROC analyses were performed using MedCalc statistical software version 15.2.2 (MedCalc Software bvba, Ostend, Belgium), and multiple and pairwise comparisons of AUCs were achieved using the DeLong nonparametric approach. The nomogram and DCA were performed using R 3.5.1 (Comprehensive R Archive Network, www.r-project.org). A significant test statistic of the calibration curve implies that the models’ prediction does not match the observed outcome perfectly [21]. The level of statistical significance was set at *P* < 0.05.

## Results

### Patients and LN characteristics

The statistical and clinical characteristics of the patients and pelvic LNs are summarized in Table 2. The prevalence rates of PLNM and non-PLNM were 26.82% (192/716) in the primary cohort and 26.06% (43/165) in the held-out cohort. No significant differences were found between the primary and held-out cohorts regarding age and the PSA level (including T-PSA, F-PSA, and F/T PSA). The median ADC value of the metastatic LNs was significantly lower than that of the nonmetastatic LNs in both the primary and held-out cohorts (primary cohort: 1.15 × 10^–3^ mm^2^/s *vs.* 1.54 × 10^–3^ mm^2^/s, *P* < 0.001; held-out cohort: 1.04 × 10^–3^ mm^2^/s *vs.* 1.38 × 10^–3^ mm^2^/s, *P* < 0.001). The short-to-long diameter ratio showed no significant difference between metastatic LNs and nonmetastatic LNs (primary cohort: 0.54 *vs.* 0.57, *P* = 0.632; 0.56 *vs.* 0.57, *P* = 0.660).

**Table 2:** Characteristics of patients in the primary and held-out cohorts

Characteristics	Primary cohort (n = 67)			Held-out cohort (n = 17)			*P* value
LN characteristics	LNM ( +)	LNM (-)	*P* value	LNM ( +)	LNM (-)	*P* value	
Number	192	716		43	165		
ADC value (10^−3^ mm^2^/s)	1.15 (0.92, 1.54)	1.54 (1.22, 2.05)	0.001	1.14 (1.08, 1.30)	1.38 (1.14, 1.75)	0.321	0.001
LN volume (cm^3^)	1.31 (0.75, 3.32)	0.41 (0.24, 0.75)	0.001	1.85 (0.90, 4.23)	0.44 (0.27, 0.80)	0.001	0.111
Short diameter (mm)	10.93 (8.99, 16.69)	7 (5.94, 8.57)	0.001	14.02 (9.13, 18.19)	7.79 (6.33, 9.12)	0.001	0.002
Long diameter (mm)	23.66 (13.06, 35.92)	10.07 (9. 21.64)	0.001	23.97 (20.41, 37.67)	14.42 (9.00, 21.51)	0.001	0.614
Short-to-long diameter ratio	0.54 (0.42, 0.67)	0.57 (0.40, 0.68)	0.632	0.56 (0.45, 0.65)	0.57 (0.40, 0.78)	0.660	0.178
*Patients characteristics*							
Age (median, IQR, y)	68 (62,74)			70 (65,72)			0.346
T-PSA (median, IQR, ng/ml)	47.16 (15.67,89.18)			35.62 (9.25, 48.64)			0.263
F-PSA (median, IQR, ng/ml)	5.17 (1.71, 11.22)			4.55 (2.08, 7.05)			0.426
F/T PSA (median, IQR, ng/ml)	0.11 (0.07, 0.18)			0.12 (0.09, 0.18)			0.486
*Gleason score [N (%)]*							
3 + 4	6 (8.96%)			1 (5.88%)			
4 + 3	14 (20.89%)			2 (11.76%)			
4 + 4	14 (20.89%)			2 (11.76%)			
4 + 5	24 (35.82%)			7 (41.18%)			
5 + 4	6 (8.96%)			3 (17.64%)			
5 + 5	3 (4.48%)			2 (11.76%)			
*Pathological T staging [N (%)]*							
pT2	20 (29.85%)			2 (11.76%)			
pT3a	11 (16.42%)			2 (11.76%)			
pT3b	22 (32.84%)			12 (70.59%)			
pT4	14 (20.89%)			1 (5.88%)			
*Clinical T staging [N (%)]*							
T2a	10 (14.93%)			–			
T2b	4 (5.97%)			–			
T2c	14 (20.89%)			3 (17.65%)			
T3a	7 (10.45%)			2(11.76%)			
T3b	15 (22.39%)			9 (52.94%)			
T4	17 (25.37%)			3 (4.48%)			

ADC = apparent diffusion coefficient; LNM = lymph nodes metastases; PSA: prostate specific antigen; F-PSA = Free PSA; T-PSA = Total PSA

### Performance of pelvic LN segmentation

**Fig. 2:**
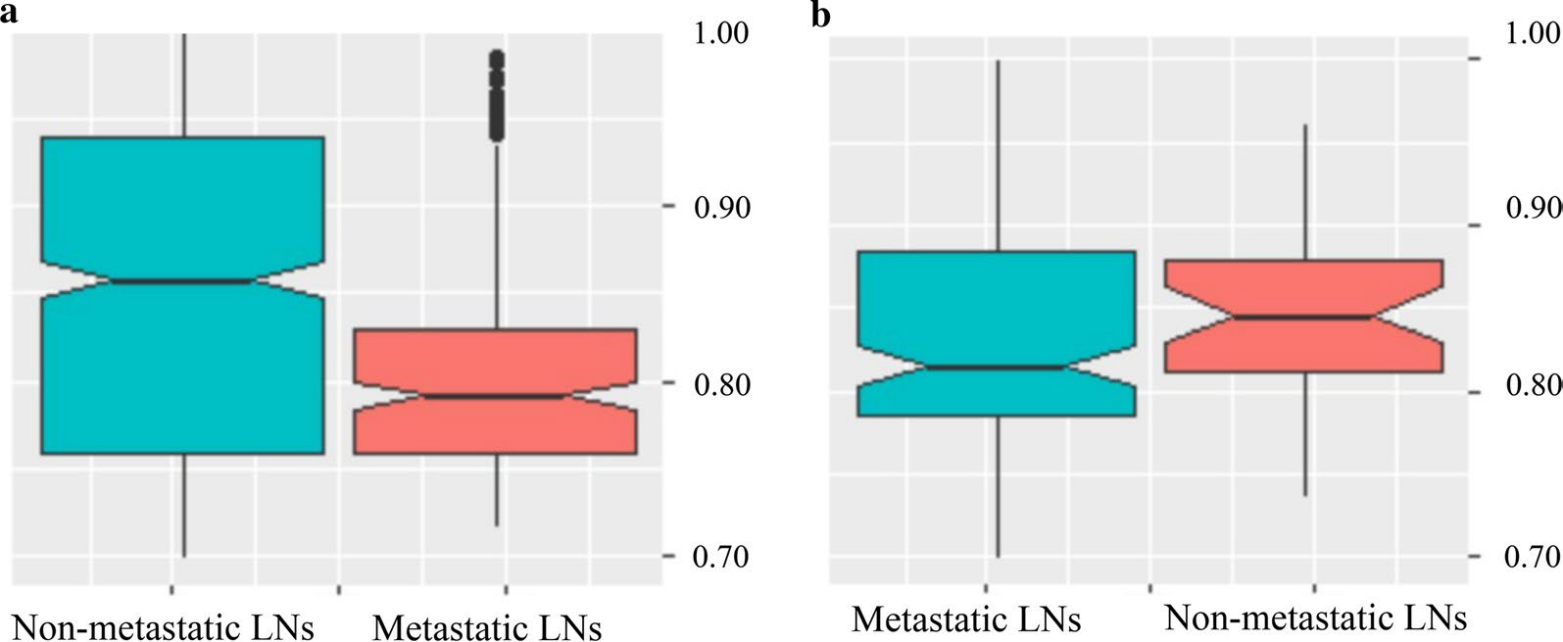
Notched box plots of the Dice scores in the primary and held-out cohort. **a** Dice scores in the primary cohort; **b** Dice scores in the held-out cohort

The Dice scores between manual and automated LNs segmentation in the primary and held-out cohort were 0.85 ± 0.09 and 0.84 ± 0.07, respectively (*P* = 0.08). The Dice score distributions in the primary and held-out cohort are shown in Fig. 2. No significant differences were found between the Dice scores of metastatic LNs and non-metastatic LNs in both primary and held-out cohorts (*P* = 0.124, 0.09) according to the notched box plots.

### Agreement of quantitative radiological characteristics

**Fig. 3:**
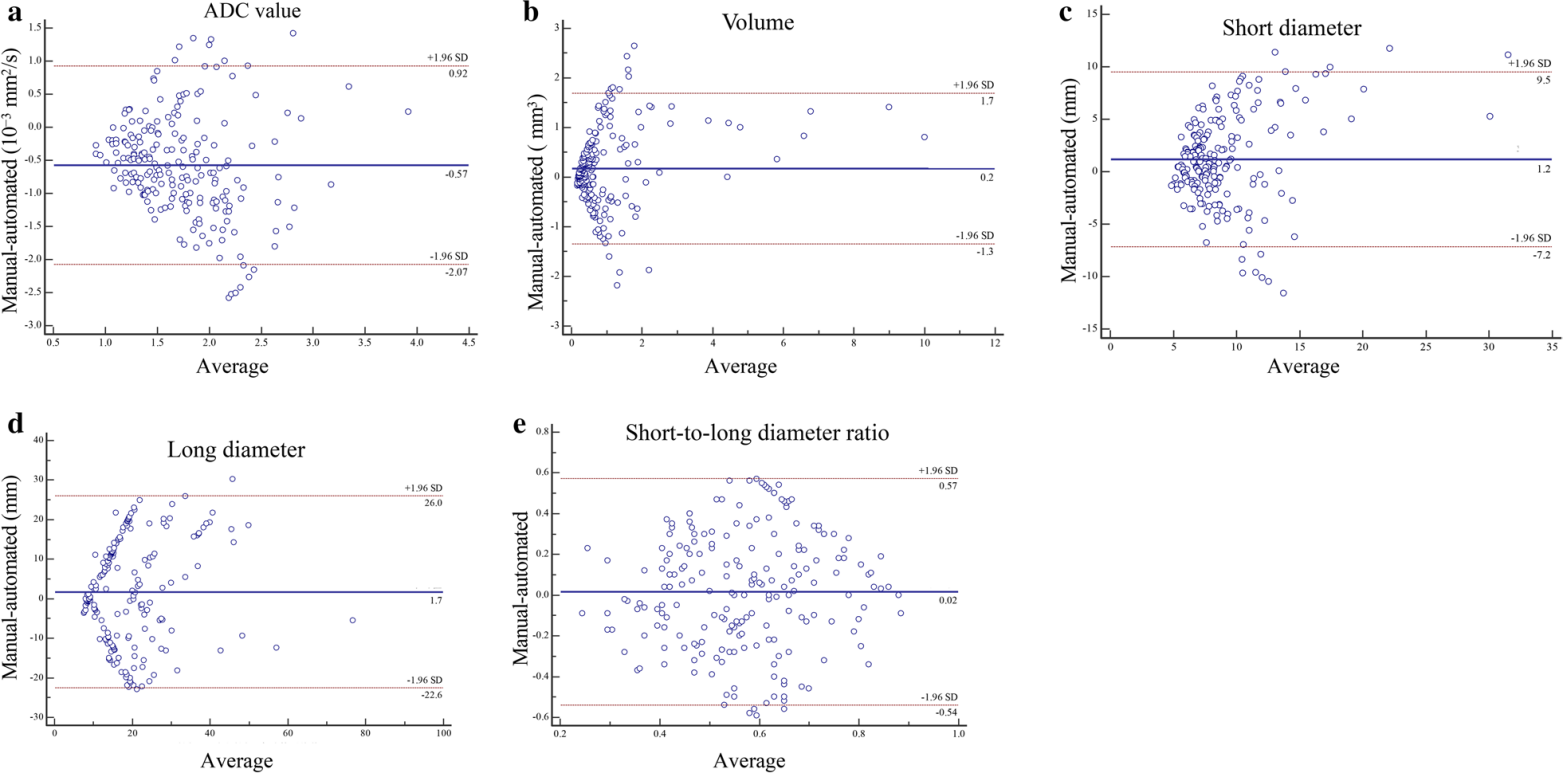
Agreement between the automatically segmented and manually segmented lymph nodes. **a** ADC values; **b** volume; **c** short diameter; **d** long diameter; **e** short-to-long diameter ratio

The agreement between the automatically segmented LNs and manually segmented LNs in terms of these quantitative radiological characteristics (ADC value, LN volume, short diameter, long diameter, and short-to-long diameter ratio) is shown in Fig. 3. The Bland–Altman analysis of the radiological features showed good consistency between the automated segmentation and manual annotation in the held-out cohort, and most values were within the consistency interval.

### Construction of Model 1 based on the quantitative radiological features

On univariate analysis, the radiological features of the pelvic LNs were compared and are summarized in Table 2. The ADC value, LN volume, short diameter and long diameter showed significant differences between the metastatic and nonmetastatic LNs and were then used to establish a radiological model to discriminate the status of the LNs using multivariate logistic analysis (Table 3). The equation to calculate the probability of metastases was generated as follows:

**Table 3:** Independent predictors of metastases in the Model 1 and Model 2

Model	Parameters	β	Odds ratio (95% CI)	*P*
Model 1	ADC value	− 0.001	(0.999,1.00)	0.001
	LN volume	0.002	(1.001, 1.003)	0.001
	Short diameter	0.264	(1.161, 1.460)	0.001
	Long diameter	− 0.137	(0.837,0.908)	0.001
	Constant	− 1.962	–	0.001
Model 2	Short diameter	0.215	(1.066, 1.442)	0.005
	LN rad-score	− 0.405	(0.003, 0.089)	0.001
	Constant	− 1.493	–	0.001

Data in parentheses are 95% confidence interval

β indicates the regression coefficient; CI = confidence interval; LN = lymph node

$$x=-1.962 -0.01\times ADC value+0.002\times LN volume+0.264\times LN short diameter-0.137\times LN long diameter$$

### Construction of Model 2 based on the radiomics and radiological features

The construction pipeline of the best model in the primary cohort was as follows: data balance: downsampling; data normalization: Z score; dimension reduction: Pearson correlation coefficient (PCC); feature selection: analysis of variance (ANOVA); classification: least absolute shrinkage and selection operator (LASSO). The detailed interpretation of the pipeline is shown in Additional file 1: S2.

Among the 1070 extracted radiomics features, after dimension reduction and feature selection, the top 10 best features for the model were selected for modeling using feature selectors (Table 4). The LN Rad-score was calculated by summing the selected features weighted by their coefficients. Multivariate logistic regression analysis of the combined radiomics model showed that a short diameter and LN Rad-score were significant risk factors for PLNM prediction (Table 3). The equation to calculate the probability of metastases was generated as follows:

**Table 4:** Key radiomics features and their coefficient

Key features	Coefficient
original_shape_Sphericity	0.856
original_shape_SurfaceVolumeRati	− 0.581
log-sigma-1-0-mm-3D_glcm_Idn	0.63
log-sigma-1-0-mm-3D_glcm_Imc1	0.096
log-sigma-1-0-mm-3D_gldm_DependenceNonUniformityNormalized	− 0.188
log-sigma-5-0-mm-3D_glcm_DifferenceEntropy	1.236
log-sigma-5-0-mm-3D_glcm_InverseVariance	− 0.139
log-sigma-5-0-mm-3D_gldm_DependenceNonUniformityNormalized	0.475
wavelet-HLL_glcm_Correlation	0.231
wavelet-HHH_glszm_SizeZoneNonUniformityNormalized	− 0.453

$$x=-1.439 -0.405\times LN Rad score+0.215\times LN short diameter$$

### PLNM discrimination performance of the prediction models

**Fig. 4:**
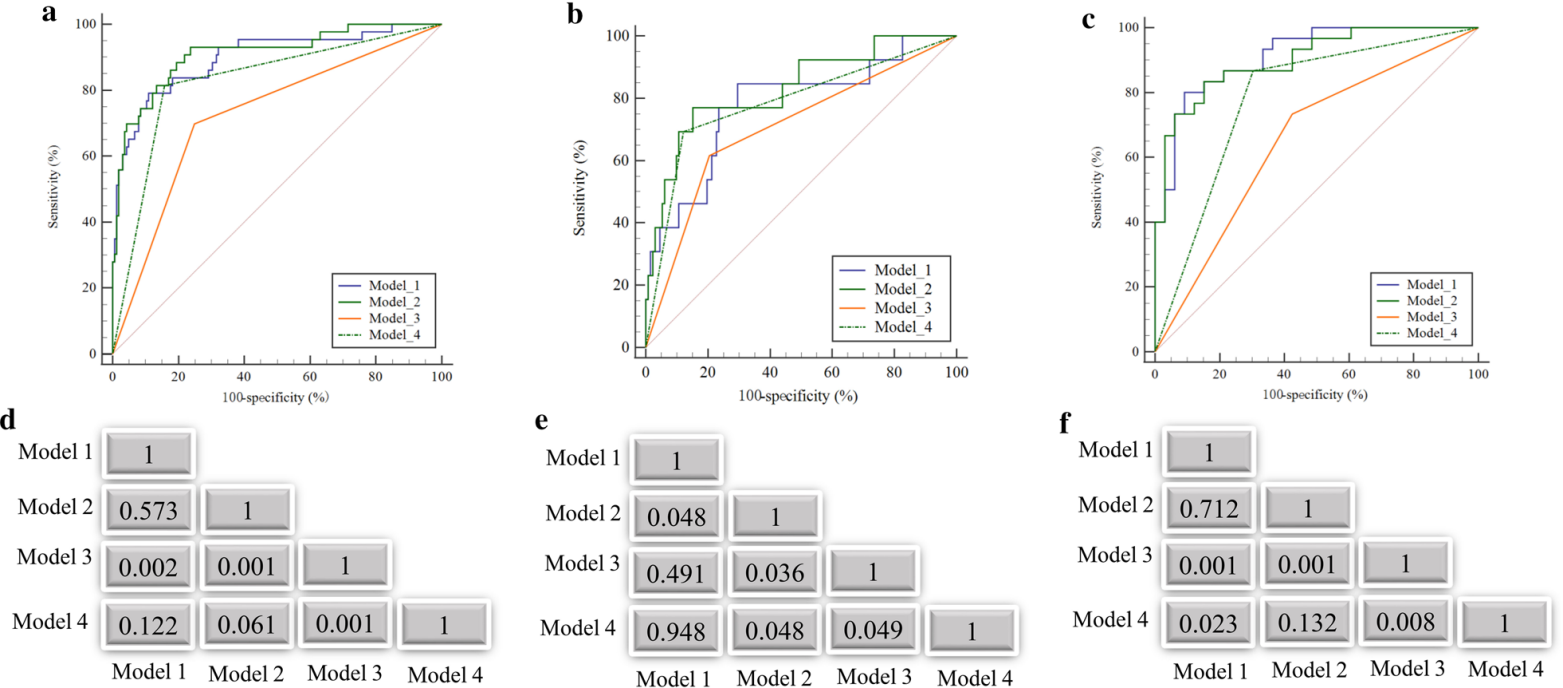
ROC curves and Delong test of the four models. **a** ROC curves of the four models in the held-out cohort, **b** LNs with short diameter ≤ 10 mm of the held-out cohort, and **c** LNs with short diameter > 10 mm of the held-out cohort. **d** Delong test of the four models in the held-out cohort, **e** LNs with short diameter ≤ 10 mm of the held-out cohort, and **f** LNs with short diameter > 10 mm of the held-out cohort

**Fig. 5:**
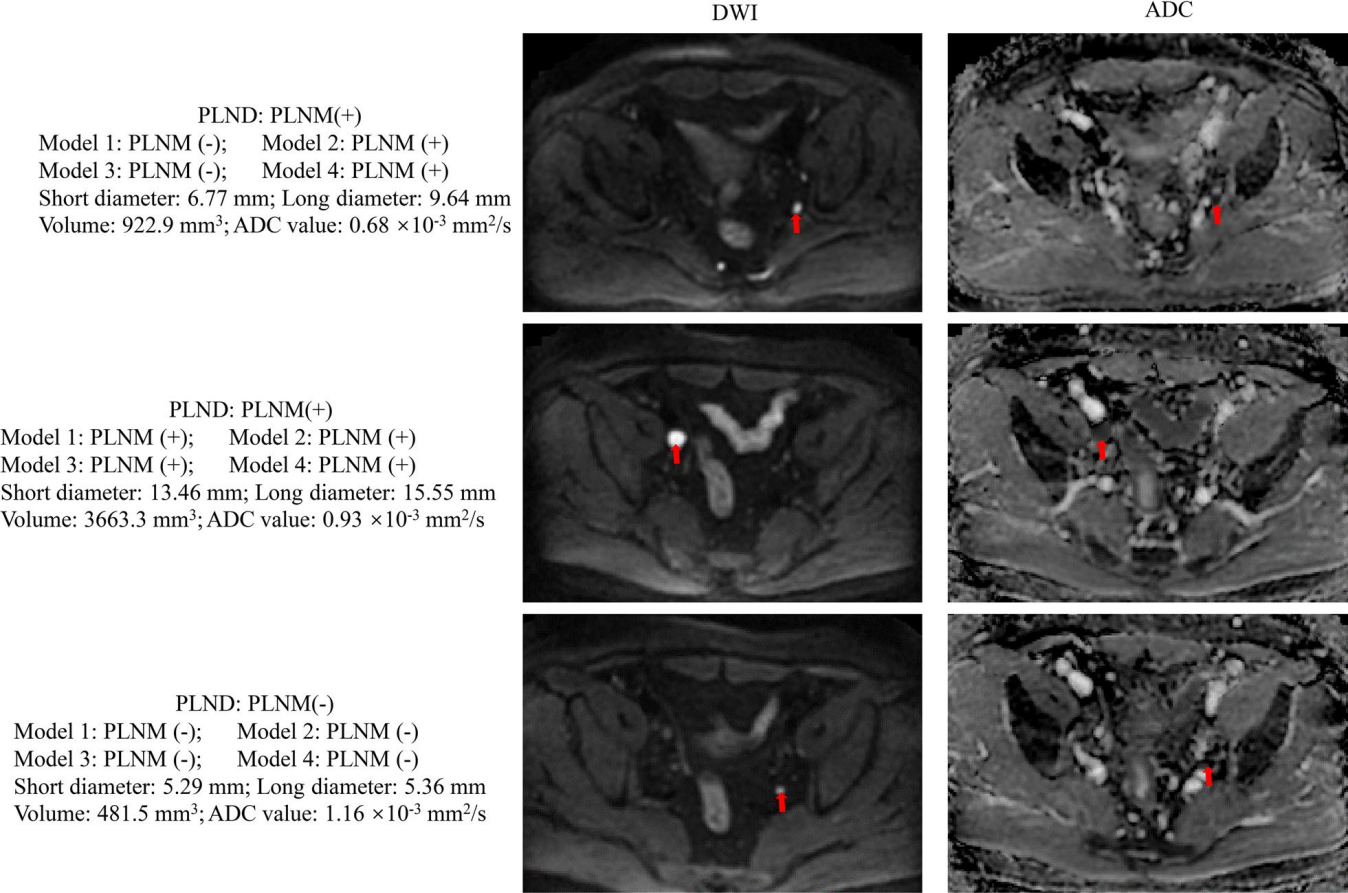
Examples for comparison of discrimination ability on PLNM. The red arrows point to the target LNs. LN: lymph node; PLND: pelvic lymph node dissection; PLNM: pelvic lymph node metastasis

The four models yielded AUCs of 0.89 (95% CI 0.85–0.94), 0.90 (95% CI 0.86–0.94), 0.71 (95% CI 0.69–0.78) and 0.78 (95% CI 0.77–0.88) in the held-out cohort for PLNM prediction (Table 5, Fig. 4a). Subgroup analysis (Fig. 4b, c) for PLNM prediction in LNs with short diameters ≤ 10 mm showed that Model 2 achieved the highest AUC (0.83; 95% CI 0.85–0.94) compared with the other models. Models 1 and 2 achieved high AUC values for PLNM prediction in the subgroup of LNs with short diameters > 10 mm, and both AUC values were significantly higher than that for Model 3 (Model 1 *vs.* Model 3: 0.91 *vs.* 0.73, *P* = 0.001; Model 2 *vs.* Model 3: 0.92 *vs.* 0.73, *P* = 0.001) according to the DeLong test (Fig. 4d–f). Example figures of PLNM prediction on different models are shown in Fig. 5.

**Table 5:** The discrimination performance of models for PLNM prediction in the held-out cohort

	AUC	ACC	SEN (%)	SPE (%)	PLR (%)	NLR (%)
Model 1	0.89 (0.85, 0.94)	0.79 (0.73, 0.85)	79.07 (64.01, 90.02)	89.09 (83.34,93.41)	7.25 (4.62, 11.51)	0.23 (0.12,0.43)
Model 2	0.90 (0.85, 0.94)	0.81 (0.75, 0.86)	88.37 (74.91, 96.12)	83.64 (77.13, 88.94)	5.40 (3.81, 7.82)	0.14 (0.06, 0.30)
Model 3	0.71 (0.69, 0.78)	0.74 (0.68, 0.79)	69.77 (53.91, 82.8)	75.15 (67.80, 81.5)	2.81 (2.01, 3.92)	0.40 (0.32, 0.61)
Model 4	0.78 (0.77, 0.88)	0.84 (0.78, 0.88)	81.40 (66.63, 91.63)	84.24 (77.80, 89.41)	5.17 (3.54, 7.62)	0.22 (0.13, 0.42)
*For LNs with short diameter ≤ 10 mm*						
Model 1	0.78 (0.70, 0.84)	0.82 (0.75, 0.88)	84.62 (54.61, 98.12)	70.45 (61.90, 78.12)	2.86 (2.01, 4.12)	0.22 (0.06, 0.82)
Model 2	0.83 (0.76, 0.89)	0.84 (0.77, 0.89)	76.92 (46.2, 95.00)	84.85 (77.6, 90.5)	5.08 (3.1, 8.4)	0.27 (0.1, 0.7)
Model 3	0.66 (0.52, 0.77)	0.65 (0.53, 0.76)	73.33 (54.12, 87.73)	57.78 (39.2, 74.5)	1.73 (1.12, 2.74)	0.46 (0.23, 0.91)
Model 4	0.74 (0.66, 0.88)	0.78 (0.66, 0.86)	86.67 (69.31, 96.25)	69.70 (51.32, 84.46)	2.86 (1.72, 4.93)	0.19 (0.07, 0.51)
*For LNs with short diameter > 10 mm*						
Model 1	0.91 (0.82, 0.97)	0.73 (0.61, 0.82)	80.00 (61.42, 92.31)	90.91 (75.73, 98.12)	8.80 (2.91, 26.32)	0.22 (0.15, 0.52)
Model 2	0.92 (0.80, 0.96)	0.81 (0.69, 0.89)	83.33 (65.3, 94.4)	84.85 (68.1, 94.9)	5.50 (2.4, 12.5)	0.2 (0.09, 0.4)
Model 3	0.73 (0.62, 0.78)	0.78 (0.70, 0.84)	61.54 (31.6, 86.1)	79.55 (71.7, 86.1)	3.01 (1.71, 5.23)	0.48 (0.22, 1.00)
Model 4	0.79 (0.71, 0.85)	0.86 (0.79, 0.91)	69.23 (38.6, 90.9)	87.88 (81.1, 92.9)	5.71 (3.2, 10.3)	0.35 (0.21, 0.83)

Data in parentheses are 95% confidence interval

AUC = area under the curve; ACC = Accuracy; SEN = Sensitivity; SPE = Specificity; PLR = Positive likelihood ratio; NLR = Negative likelihood ratio; PLNM = pelvic lymph node metastases

In addition, at the patient level, Model 1, Model 2 and Model 4 discriminated all 17 patients as positive PLNM in the held-out cohort (sensitivity: 100%), while two patients with positive PLNM were wrongly taken as negative by Model 1 (sensitivity: 88.24%).

### Clinical usefulness of Model 1 and Model 2

**Fig. 6:**
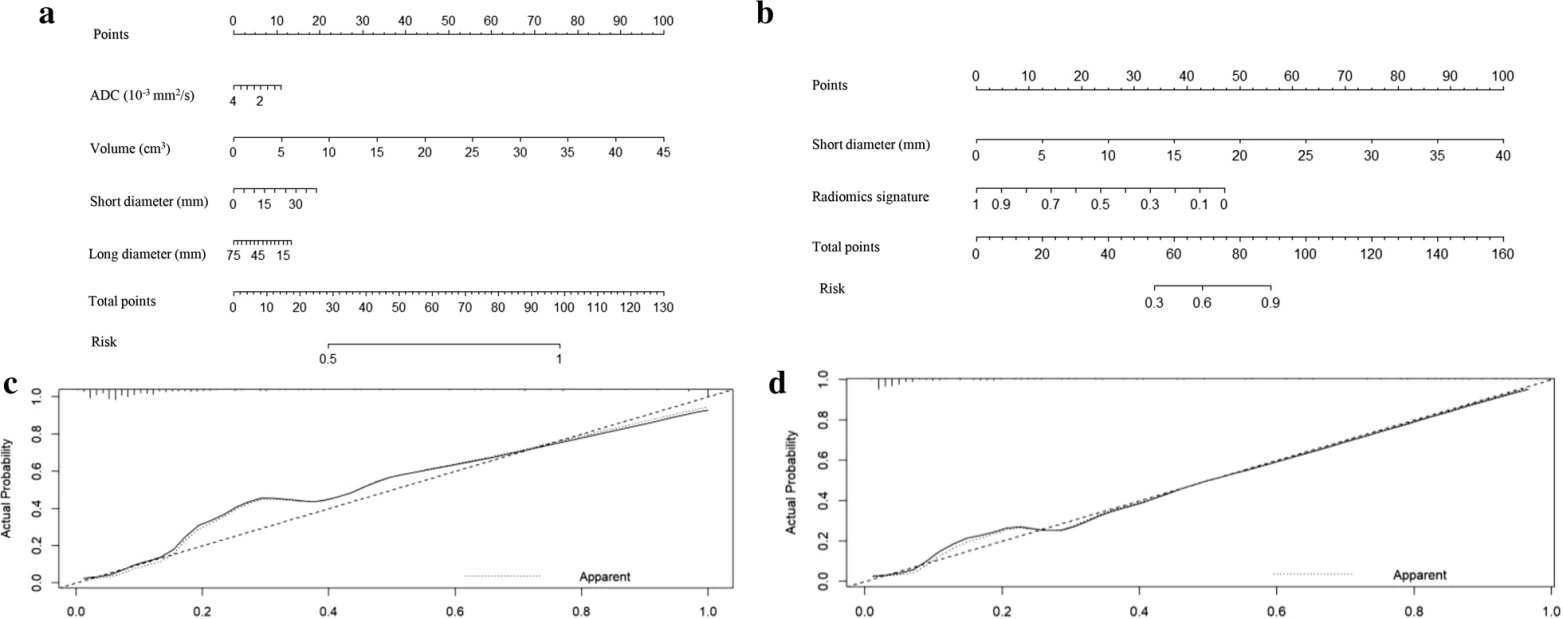
Nomograms and calibration curves in the held-out cohort. **a** The radiological nomogram of Model 1 integrated only quantitative radiological factors. **b** The radiomics nomogram of Model 2 integrated radiological factors with the radiomics signature. **c** The calibration curve of Model 1 based on the quantitative radiological features. **d** The calibration curve of Model 2 based on the radiomics and radiological features

The nomograms of Models 1 and 2 yielded C-index values of 0.804 and 0.910, respectively, in the held-out cohort. The nomograms and calibration curves of Models 1 and 2 are shown in Fig. 6. The Hosmer–Lemeshow test showed a nonsignificant statistic (*P* = 0.075 and *P* = 0.088, respectively) for Models 1 and 2, demonstrating no significant deviation between the calibration curve and a perfect fit for PLNM prediction.

### Clinical benefit of the prediction models

**Fig. 7:**
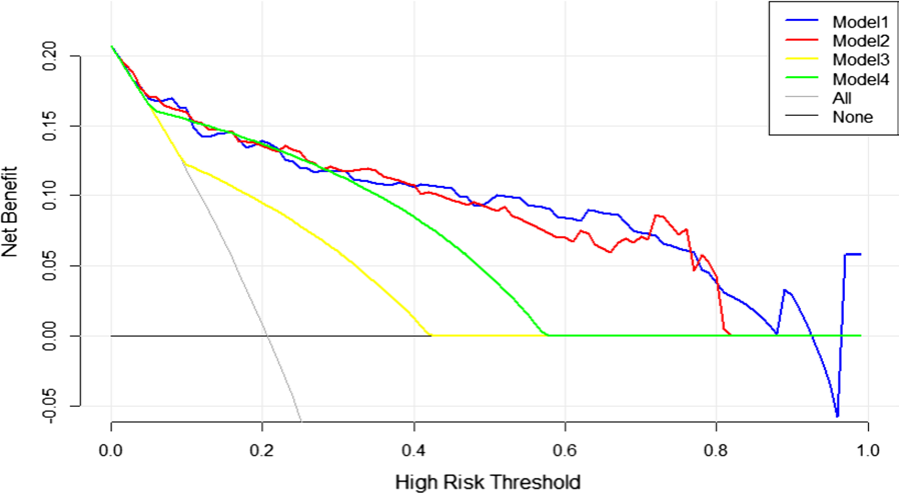
Decision curve analysis comparing the net benefits of the four model

The DCA curves of the four models for PLNM prediction are presented in Fig. 7. All the models obtained higher net benefits than the PLNM-all or PLNM-none protocol in different ranges of threshold probabilities. If the risk threshold probability is set over 35%, Models 1 and 2 have more advantages to predict PLNM than Models 3 and 4.

## Discussion

In this retrospective study, we constructed two prediction models for PLNM based on automatic LN segmentation with quantitative radiological LN features alone (Model 1: ADC value, LN volume, short diameter and long diameter) and the combination of quantitative radiological features and radiomics signatures (Model 2: short diameter and LN Rad-score) via multiple logistic regression. Our results showed no significant difference between the AUCs of Models 1 and 2 [0.89 (95% CI 0.85, 0.94) *vs.* 0.90 (95% CI 0.85, 0.94), *P* = 0.573) in the held-out cohort. Considering that the size, particularly the short diameter, of the LNs is an important factor influencing the performance of the model for LN prediction, all the LNs were divided into two subgroups according to a threshold of 10 mm [17, 22]. Regarding the subgroup of LNs with a short diameter ≤ 10 mm of the held-out cohort, Model 2 showed a higher AUC than Model 1 [0.83 (95% CI 0.76, 0.89) *vs.* 0.78 (95% CI 0.70, 0.84), *P* = 0.048]. Therefore, the prediction model of the combination of the radiomics signature and radiological features demonstrated better predictive efficacy than the radiological factors alone, indicating that the predictive model could be a better tool to predict PLNM in PCa patients.

MRI has been widely used as a noninvasive imaging modality to evaluate the LN status with mixed results because the diagnostic accuracy of LN metastases depends largely on the level and experience of the radiologists [23]. As a functional imaging technique, DWI enables the noninvasive characterization of biological tissues based on the random translational molecular motion of water molecules. The degree of diffusion restriction can be quantitatively expressed by calculating ADC maps, allowing tissue characterization [10, 24]. Several studies have reported significant differences between the ADC values in benign and malignant LNs, yielding high accuracy to differentiate between malignant and benign LNs [25, 26]. Fewer promising results were shown in other studies. For example, Thoeny et al. reported nonsignificant findings in the ADC values of metastatic and benign LNs, which were (0.94 ± 0.18) × 10^−3^ mm^2^/s and (1.01 ± 0.28) × 10^−3^ mm^2^/s, respectively [27]. Therefore, although a trend exists toward a lower mean ADC value in metastatic LNs, the role of ADC in the assessment of nodal status remains debatable.

Size is usually considered a fundamental criterion to diagnose nodal metastases. Various benign LN sizes can substantially overlap with the size of metastatic nodes, resulting in the suboptimal sensitivity and specificity profile of size criteria [28]. The shape of LNs can also be a helpful diagnostic feature. A normal LN has a fatty hilum and is an oblong kidney-bean-shaped structure, and a higher short-to-long-axis ratio (rounder than oblong) is more likely to be malignant [17]. In a study of patients with cervical cancer, the short-to-long ratio was not found to be a significant factor to differentiate metastatic and nonmetastatic LNs [29]. Given the limited accuracy of any of these features considered alone, using a combination of size, shape and ADC value criteria together seems prudent.

In this study, we built a multivariate model based on quantitative radiological features for PLNM prediction. Patterns of the LN short diameter, long diameter, ADC value and volume were finally included through multivariate logistic analysis. The AUC value of the model was 0.89 (95% CI 0.85, 0.94) in the held-out cohort but was relatively lower in the subgroup of LNs with short diameters ≤ 10 mm [0.78 (95% CI 0.70, 0.84)]. These normal-sized LNs represent the most challenging problem in clinical practice. Therefore, the diagnostic performance of normal-sized LNs must be improved.

Considering that radiomics features comprise quantitative and detailed information in multiple dimensions and could reflect the heterogeneity and biological behavior of metastases [30], we hypothesized that radiomics could provide more information to distinguish metastases that are challenging for traditional radiologic interpretations. In this study, ten radiomics features were included—2 shape features, 5 Gy level cooccurrence matrix (GLCM) features, 1 Gy level size zone matrix (GLSZM), and 2 Gy level dependence matrix (GLDM) features. Only features closely correlated with the LN status were selected for redundancy elimination by narrowing their regression coefficients based on the widely used LASSO algorithm. This proposed algorithm could identify wide associations between the extracted radiomics data and construct a robust radiomics signature with a panel of selected steady variables. This approach not only effectively identifies important radiomics features but also avoids the overfitting problem for the classification task [31, 32].

In this research, the model with the highest AUC value for PLNM prediction was finally regarded as the best model. Downsampling was selected as the method of data balance in the pipeline of the best model, which eliminated the impact of data imbalance on model development by by sampling the signal at a lower rate [33]. PCC and ANOVA were used to reduce the dimensions of the feature matrix and to select the best features, respectively [34]. LASSO is a popular penalized regression method that minimizes the residual sum of squares and places a bound on the sum of the absolute value of the coefficients [35].

Previous studies on PLNM prediction have shown that radiomics features combined with clinical and/or radiological features enable superior prediction ability [29, 36]. Therefore, in this study, Model 2 was constructed by combining the radiological features and the LN Rad-score using multivariate logistic regression. Because some radiological features might be covered by the radiomics signatures, radiological factors such as the ADC value and LN volume were no longer independent predictors when multifactor regression was performed.

Our results showed that Model 2 improved the discrimination ability of PLNM compared with Model 1, particularly for normal-sized LNs. An explanation might be that normal-sized metastatic LNs have a higher probability of being in the early phase, and the metastatic foci inside the LNs may be very tiny or even at the cellular level. These changes may be challenging to detect by MRI. Additionally, the high C-index of the nomogram analysis for Model 2 further confirmed its reliability and clinical usage. This radiomics nomogram model is promising as a visualized and easy-to-use tool for preoperative PLNM and helps clinically make individualized treatment decisions in patients with PCa.

Several studies have demonstrated the potential benefit of radiomics nomograms to predict PLNM in PCa [37, 38]. Almost all of these studies were conducted at the patient level. The main reason is that a node-by-node correspondence between DWI and histopathology could be challenging to obtain. In this study, we applied carefully matched one-to-one MR-pathologically confirmed LNs as the standard reference for PLNM prediction according to the ePLND/PLND results. All the labeled LNs were within the dissection region, and LNs with an uncertain status were neglected.

Additionally, comparing the visual assessments of the junior radiologists (Model 3) and senior radiologists (Model 4), we found that the AUC value of Model 2 was superior to that of the junior radiologists [AUC: 0.90 (95% CI 0.85, 0.94) *vs.* 0.71 (95% CI 0.69, 0.78), *P* = 0.001] and equivalent to that of senior radiologists [AUC: 0.90 (95% CI 0.85, 0.94) *vs.* 0.78 (95% CI 0.77, 0.88), *P* = 0.061] in the held-out cohort. For the subgroup of LNs with short diameters ≤ 10 mm of the held-out cohort, Model 2 achieved significantly higher discrimination ability than senior radiologists [AUC: 0.83 (95% CI 0.76, 0.89) *vs.* 0.74 (95% CI 0.66, 0.88), *P* = 0.048], demonstrating that our proposed radiomics approach could also promisingly provide an outperformed prediction performance for PLNM compared with the visual assessments of the radiologists.

Some practical issues should be considered when applying radiomics in the clinic, such as time and labor resources. VOI acquisition is a critical but time-consuming job that usually presents an obstacle for radiologists to perform radiomics analysis [39]. In this study, we applied a pretrained deep learning approach that enables rapid and accurate detection and segmentation of LNs on DWI images in the setting of PCa N-staging. Based on automatic nodal staging, Model 2 achieved excellent performance in PLNM prediction concerning accuracy, sensitivity, and specificity.

We acknowledge limitations to our study. First, this study was retrospective with limited sample size, and extending the primary cohort to more patients might further promote the performance of the radiomics model for clinical application. Second, in this study, all the data in the primary and held-put cohorts were collected from a single institution, therefore the application and performance of the model in other institutions remains unclear until now. Multi-institution validation is vital for model application in practice. Third, we did not compare or combine our results with radiomics analysis of other MRI sequences, such as T2WI or DCE, which might improve the diagnostic efficiency. We will compare the diagnostic efficiency in future research. Fourth, the sample size of positive LNM and negative LNM was imbalanced in the primary cohort (192:716). Although preprocessed down-sampling was performed for data balance, the influence of this operation on the diagnostic performance was not ensured. Finally, deep learning models have been shown to perform relatively well in many tasks and might outperform radiomics models [40]. Developing a prediction model based on deep learning and comparing its performance with the current study may be necessary for the future.

In conclusion, the noninvasive LN radiomics model based on the quantitative radiological features and radiomics signature in our study can achieve accurate PLNM prediction based on DWI preoperatively. A key advantage of this study is indicated by the result that the combined radiomics model has more predictive efficacy than senior radiologists for differentiating malignant and benign normal-sized PLNM, a finding that could be helpful for patients with PCa to optimize decision-making and adjust adjuvant treatments.

## Supplementary Information

**Additional file 1.** **S1:** Radiomics feature extraction. **Table S1:** Radiomic features used in this study. **Table S2:** Available options for each step in the radiomics model development pipeline. **S2:** Details of the radiomics modeling pipelines.

## Data Availability

The datasets used and/or analyzed during the current study are available from the corresponding author on reasonable request.

## Abbreviations

ADC: Apparent diffusion coefficient
AUC: Area under the curve
CI: Confidence internal
DCA: Decision curve analysis
DCE: Dynamic contrast-enhanced
DWI: Diffusion-weighted imaging
EAU: European Association of Urology
ePLND: Extended pelvic lymph node dissection
fT2WI: T2-weighted images with fat suppression
LN: Lymph node
mpMRI: Multiparametric magnetic resonance imaging
NLR: Negative likelihood ratio
PCa: Prostate cancer
PLNM: Pelvic lymph node metastasis
PLR: Positive likelihood ratio
PSA: Prostate-specific antigen
ROC: Receiver operating characteristic curve
RP: Radical prostatectomy
T1WI: T1-weighted images
VOI: Volumes of interest

## Code availability

The code used for the development of the algorithm are available from the corresponding author on reasonable request.

## References

[1] Mottet N, Bellmunt J, Bolla M, Briers E, Cumberbatch MG, De Santis M, Fossati N, Gross T, Henry AM, Joniau S (2017). EAU-ESTRO-SIOG guidelines on prostate cancer. Part 1: screening, diagnosis, and local treatment with curative intent. Eur Urol.

[2] Fossati N, Willemse PM, Van den Broeck T, van den Bergh RCN, Yuan CY, Briers E, Bellmunt J, Bolla M, Cornford P, De Santis M (2017). The benefits and harms of different extents of lymph node dissection during radical prostatectomy for prostate cancer: a systematic review. Eur Urol.

[3] Costello AJ (2020). Considering the role of radical prostatectomy in 21st century prostate cancer care. Nat Rev Urol.

[4] van Leeuwen FWB, Winter A, van Der Poel HG, Eiber M, Suardi N, Graefen M, Wawroschek F, Maurer T (2019). Technologies for image-guided surgery for managing lymphatic metastases in prostate cancer. Nat Rev Urol.

[5] Yaxley JW, Dagher J, Delahunt B, Egevad L, Srigley J, Samaratunga H (2018). Reconsidering the role of pelvic lymph node dissection with radical prostatectomy for prostate cancer in an era of improving radiological staging techniques. World J Urol.

[6] Pierorazio PM, Gorin MA, Ross AE, Feng Z, Trock BJ, Schaeffer EM, Han M, Epstein JI, Partin AW, Walsh PC (2013). Pathological and oncologic outcomes for men with positive lymph nodes at radical prostatectomy: the Johns Hopkins Hospital 30-year experience. Prostate.

[7] Marcus DM, Rossi PJ, Nour SG, Jani AB (2014). The impact of multiparametric pelvic magnetic resonance imaging on risk stratification in patients with localized prostate cancer. Urology.

[8] Morote J, Celma A, Roche S, de Torres IM, Mast R, Semedey ME, Regis L, Planas J (2019). Who benefits from multiparametric magnetic resonance imaging after suspicion of prostate cancer?. European urology oncology.

[9] Fortuin A, Rooij M, Zamecnik P, Haberkorn U, Barentsz J (2013). Molecular and functional imaging for detection of lymph node metastases in prostate cancer. Int J Mol Sci.

[10] Eiber M, Beer AJ, Holzapfel K, Tauber R, Ganter C, Weirich G, Krause BJ, Rummeny EJ, Gaa J (2010). Preliminary results for characterization of pelvic lymph nodes in patients with prostate cancer by diffusion-weighted MR-imaging. Invest Radiol.

[11] Hövels AM, Heesakkers RA, Adang EM, Jager GJ, Strum S, Hoogeveen YL, Severens JL, Barentsz JO (2008). The diagnostic accuracy of CT and MRI in the staging of pelvic lymph nodes in patients with prostate cancer: a meta-analysis. Clin Radiol.

[12] Mayerhoefer ME, Materka A, Langs G, Häggström I, Szczypiński P, Gibbs P, Cook G (2020). Introduction to radiomics. J Nucl Med.

[13] Sun Y, Reynolds HM, Parameswaran B, Wraith D, Finnegan ME, Williams S, Haworth A (2019). Multiparametric MRI and radiomics in prostate cancer: a review. Australas Phys Eng Sci Med.

[14] Kumar V, Gu Y, Basu S, Berglund A, Eschrich SA, Schabath MB, Forster K, Aerts HJ, Dekker A, Fenstermacher D (2012). Radiomics: the process and the challenges. Magn Reson Imaging.

[15] Avanzo M, Wei L, Stancanello J, Vallières M, Rao A, Morin O, Mattonen SA, El Naqa I (2020). Machine and deep learning methods for radiomics. Med Phys.

[16] Park JE, Kickingereder P, Kim HS (2020). Radiomics and deep learning from research to clinical workflow: neuro-oncologic imaging. Korean J Radiol.

[17] McMahon CJ, Rofsky NM, Pedrosa I (2010). Lymphatic metastases from pelvic tumors: anatomic classification, characterization, and staging. Radiology.

[18] Liu X, Sun Z, Han C, Cui Y, Huang J, Wang X, Zhang X, Wang X (2021). Development and validation of the 3D U-Net algorithm for segmentation of pelvic lymph nodes on diffusion-weighted images. BMC Med Imaging.

[19] Song Y, Zhang J, Zhang YD, Hou Y, Yan X, Wang Y, Zhou M, Yao YF, Yang G (2020). FeAture explorer (FAE): a tool for developing and comparing radiomics models. PLoS ONE.

[20] Vickers AJ, van Calster B, Steyerberg EW (2019). A simple, step-by-step guide to interpreting decision curve analysis. Diagnost Prognost Res.

[21] Kramer AA, Zimmerman JE (2007). Assessing the calibration of mortality benchmarks in critical care: the Hosmer-Lemeshow test revisited. Crit Care Med.

[22] Woo S, Suh CH, Kim SY, Cho JY, Kim SH (2018). The Diagnostic performance of MRI for detection of lymph node metastasis in bladder and prostate cancer: an updated systematic review and diagnostic meta-analysis. AJR Am J Roentgenol.

[23] Bedrikovetski S, Dudi-Venkata NN, Maicas G, Kroon HM, Seow W, Carneiro G, Moore JW, Sammour T (2021). Artificial intelligence for the diagnosis of lymph node metastases in patients with abdominopelvic malignancy: a systematic review and meta-analysis. Artif Intell Med.

[24] Caglic I, Barrett T (2018). Diffusion-weighted imaging (DWI) in lymph node staging for prostate cancer. Transl Androl Urol.

[25] Roy C, Bierry G, Matau A, Bazille G, Pasquali R (2010). Value of diffusion-weighted imaging to detect small malignant pelvic lymph nodes at 3 T. Eur Radiol.

[26] Beer AJ, Eiber M, Souvatzoglou M, Holzapfel K, Ganter C, Weirich G, Maurer T, Kübler H, Wester HJ, Gaa J (2011). Restricted water diffusibility as measured by diffusion-weighted MR imaging and choline uptake in (11)C-choline PET/CT are correlated in pelvic lymph nodes in patients with prostate cancer. Mol Imag Biol.

[27] Thoeny HC, Froehlich JM, Triantafyllou M, Huesler J, Bains LJ, Vermathen P, Fleischmann A, Studer UE (2014). Metastases in normal-sized pelvic lymph nodes: detection with diffusion-weighted MR imaging. Radiology.

[28] Brown G, Richards CJ, Bourne MW, Newcombe RG, Radcliffe AG, Dallimore NS, Williams GT (2003). Morphologic predictors of lymph node status in rectal cancer with use of high-spatial-resolution MR imaging with histopathologic comparison. Radiology.

[29] Song J, Hu Q, Ma Z, Zhao M, Chen T, Shi H (2021). Feasibility of T(2)WI-MRI-based radiomics nomogram for predicting normal-sized pelvic lymph node metastasis in cervical cancer patients. Eur Radiol.

[30] Aerts HJ, Velazquez ER, Leijenaar RT, Parmar C, Grossmann P, Carvalho S, Bussink J, Monshouwer R, Haibe-Kains B, Rietveld D (2014). Decoding tumour phenotype by noninvasive imaging using a quantitative radiomics approach. Nat Commun.

[31] Hepp T, Schmid M, Gefeller O, Waldmann E, Mayr A (2016). Approaches to regularized regression - a comparison between gradient boosting and the lasso. Methods Inf Med.

[32] Ma S, Xie H, Wang H, Han C, Yang J, Lin Z, Li Y, He Q, Wang R, Cui Y (2019). MRI-based radiomics signature for the preoperative prediction of extracapsular extension of prostate cancer. J Magn Reson Imaging.

[33] Zhu S, He Z, Meng X, Meng X, Zhou J, Guo Y, Zeng B: A new polyphase down-sampling based multiple description image coding. IEEE Trans Image Process 2020. 10.1109/TIP.2020.2984876 32275591

[34] Lin YC, Lin CH, Lu HY, Chiang HJ, Wang HK, Huang YT, Ng SH, Hong JH, Yen TC, Lai CH (2020). Deep learning for fully automated tumor segmentation and extraction of magnetic resonance radiomics features in cervical cancer. Eur Radiol.

[35] Ma X, Mo C, Huang L, Cao P, Shen L, Gui C (2021). An robust rank aggregation and least absolute shrinkage and selection operator analysis of novel gene signatures in dilated cardiomyopathy. Front Cardiovasc Med.

[36] Wang T, Gao T, Yang J, Yan X, Wang Y, Zhou X, Tian J, Huang L, Zhang M (2019). Preoperative prediction of pelvic lymph nodes metastasis in early-stage cervical cancer using radiomics nomogram developed based on T2-weighted MRI and diffusion-weighted imaging. Eur J Radiol.

[37] Gandaglia G, Ploussard G, Valerio M, Mattei A, Fiori C, Fossati N, Stabile A, Beauval JB, Malavaud B, Roumiguié M (2019). A novel nomogram to identify candidates for extended pelvic lymph node dissection among patients with clinically localized prostate cancer diagnosed with magnetic resonance imaging-targeted and systematic biopsies. Eur Urol.

[38] Gandaglia G, Martini A, Ploussard G, Fossati N, Stabile A, De Visschere P, Borgmann H, Heidegger I, Steinkohl F, Kretschmer A (2020). External validation of the 2019 briganti nomogram for the identification of prostate cancer patients who should be considered for an extended pelvic lymph node dissection. Eur Urol.

[39] Han C, Ma S, Liu X, Liu Y, Li C, Zhang Y, Zhang X, Wang X: Radiomics models based on apparent diffusion coefficient maps for the prediction of high-grade prostate cancer at radical prostatectomy: comparison with preoperative biopsy. J Magn Reson Imaging 2021. 10.1002/jmri.27565 33682286

[40] Debats OA, Litjens GJ, Barentsz JO, Karssemeijer N, Huisman HJ (2011). Automated 3-dimensional segmentation of pelvic lymph nodes in magnetic resonance images. Med Phys.

