# Supplementary material for: Utility of diffusion weighted imaging-based radiomics nomogram to predict pelvic lymph nodes metastasis in prostate cancer
Source: BMC Med Imaging. 2022 Nov 4;22:190. doi: 10.1186/s12880-022-00905-3 (PMC9636642; doi:10.1186/s12880-022-00905-3)
Supplement: Supplementary file 1 — Additional file 1. S1: Radiomics feature extraction. Table S1: Radiomic features used in this study. Table S2: Available options for each step in the radiomics model development pipeline. S2: Details of the radiomics modeling pipelines. [file 12880_2022_905_MOESM1_ESM.docx]

**Supplementary Materials**

***S1*: Radiomics feature extraction**

To extract the radiomics features, the following 3 steps related to the parameter setting were performed (1):

1. Three settings are specified as follows: “bin Width”, “label”, and “voxel Array Shift”.

2. Three image types are enabled (“Original”, “LoG” (Laplacian of Gaussian) and “Wavelet”), with custom settings specified for “LoG” (“sigma: [1.0, 3.0, 5.0]”) and “Wavelet” (“[LLH, LHL, HLL, LHH, HHL, HLH, HHH, LLL]”).

3. The following six feature classes are defined: “shape”, “first-order”, “glcm”, “glrlm”, “glszm” and “gldm”; the classes are enabled with all possible features in the respective class.

The following three groups of radiomics features were used (2): first-order statistical features (n=18), shape-based features (n=14), and textural features (n=24 GLCM +16 GLRLM +16 GLSZM +14 GLDM) (S2).

Therefore, 216 ((1 Original + 3 LoG + 8 Wavelet) ×18) first-order statistical features, 14 shape-based features and 840 ((1 Original + 3 LoG + 8 Wavelet) × (24+16+16+14)) texture features (for a total of 1070 (216+14+840) radiomics features) were extracted from DWI.

***Table S1*: Radiomic features used in this study.**

|  |  | Features | |
| --- | --- | --- | --- |
| **First-order Statistical Features** |  | 10^th^ Percentile | |
| **(n=18)** |  | 90^th^ Percentile | |
|  |  | Energy | |
|  |  | Entropy | |
|  |  | Interquartile Range | |
|  |  | Kurtosis | |
|  |  | Maximum | |
|  |  | Mean Absolute Deviation | |
|  |  | Mean | |
|  |  | Median | |
|  |  | Minimum | |
|  |  | Range | |
|  |  | Robust Mean Absolute Deviation | |
|  |  | Root Mean Squared | |
|  |  | Skewness | |
|  |  | Total Energy | |
|  |  | Uniformity | |
|  |  | Variance | |
| **Shape Features** |  | Elongation |  |
| **(n=14)** |  | Flatness | |
|  |  | Least Axis Length | |
|  |  | Major Axis Length | |
|  |  | Maximum 2D Diameter (Column) | |
|  |  | Maximum 2D Diameter (Row) | |
|  |  | Maximum 2D Diameter (Slice) | |
|  |  | Maximum 3D Diameter | |
|  |  | Mesh Volume | |
|  |  | Minor Axis Length | |
|  |  | Sphericity | |
|  |  | Surface Area | |
|  |  | Surface Volume Ratio | |
|  |  | Voxel Volume | |
| **Textural Features: Gray Level Co-occurrence Matrix (GLCM) Features** |  | Autocorrelation | |
| **(n=24)** |  | Cluster Prominence | |
|  |  | Cluster Shade | |
|  |  | Cluster Tendency | |
|  |  | Contrast | |
|  |  | Correlation | |
|  |  | Difference Average | |
|  |  | Difference Entropy | |
|  |  | Difference Variance | |
|  |  | Inverse Difference (ID) | |
|  |  | Inverse Difference Moment (IDM) | |
|  |  | Inverse Difference Moment Normalized (IDMN) | |
|  |  | Inverse Difference Normalized (IDN) | |
|  |  | Informational Measure of Correlation (IMC) 1 | |
|  |  | Informational Measure of Correlation (IMC) 2 | |
|  |  | Inverse Variance | |
|  |  | Joint Average | |
|  |  | Joint Energy | |
|  |  | Joint Entropy | |
|  |  | Maximal Correlation Coefficient (MCC) | |
|  |  | Maximum Probability | |
|  |  | Sum Average | |
|  |  | Sum Entropy | |
|  |  | Sum of Squares | |
| **Textural Features: Gray Level Run Length Matrix (****GLRLM) Features** |  | Gray Level Non-Uniformity (GLN) | |
| **(n=16)** |  | Gray Level Non-Uniformity Normalized (GLNN) | |
|  |  | Gray Level Variance (GLV) | |
|  |  | High Gray Level Run Emphasis (HGLRE) | |
|  |  | Long Run Emphasis (LRE) | |
|  |  | Long Run High Gray Level Emphasis (LRHGLE) | |
|  |  | Long Run Low Gray Level Emphasis (LRLGLE) | |
|  |  | Low Gray Level Run Emphasis (LGLRE) | |
|  |  | Run Entropy (RE) | |
|  |  | Run Length Non-Uniformity (RLN) | |
|  |  | Run Length Non-Uniformity Normalized (RLNN) | |
|  |  | Run Percentage (RP) | |
|  |  | Run Variance (RV) | |
|  |  | Short Run Emphasis (SRE) | |
|  |  | Short Run High Gray Level Emphasis (SRHGLE) | |
|  |  | Short Run Low Gray Level Emphasis (SRLGLE) | |
| **Textural Features: Gray Level Size Zone Matrix (****GLSZM) Features** |  | Gray Level Non-Uniformity (GLN) | |
| **(n=16)** |  | Gray Level Non-Uniformity Normalized (GLNN) | |
|  |  | Gray Level Variance (GLV) | |
|  |  | High Gray Level Zone Emphasis (HGLZE) | |
|  |  | Large Area Emphasis (LAE) | |
|  |  | Large Area High Gray Level Emphasis (LAHGLE) | |
|  |  | Large Area Low Gray Level Emphasis (LALGLE) | |
|  |  | Low Gray Level Zone Emphasis (LGLZE) | |
|  |  | Size-Zone Non-Uniformity (SZN) | |
|  |  | Size-Zone Non-Uniformity Normalized (SZNN) | |
|  |  | Small Area Emphasis (SAE) | |
|  |  | Small Area High Gray Level Emphasis (SAHGLE) | |
|  |  | Small Area Low Gray Level Emphasis (SALGLE) | |
|  |  | Zone Entropy (ZE) | |
|  |  | Zone Percentage (ZP) | |
|  |  | Zone Variance (ZV) | |
| **Textural Features: Gray Level Dependence Matrix (****GLDM) Features** |  | Dependence Entropy (DE) | |
| **(N=14)** |  | Dependence Non-Uniformity (DN) | |
|  |  | Dependence Non-Uniformity Normalized (DNN) | |
|  |  | Dependence Variance (DV) | |
|  |  | Gray Level Non-Uniformity (GLN) | |
|  |  | Gray Level Variance (GLV) | |
|  |  | High Gray Level Emphasis (HGLE) | |
|  |  | Large Dependence Emphasis (LDE) | |
|  |  | Large Dependence High Gray Level Emphasis (LDHGLE) | |
|  |  | Large Dependence Low Gray Level Emphasis (LDLGLE) | |
|  |  | Low Gray Level Emphasis (LGLE) | |
|  |  | Small Dependence Emphasis (SDE) | |
|  |  | Small Dependence High Gray Level Emphasis (SDHGLE) | |
|  |  | Small Dependence Low Gray Level Emphasis (SDLGLE) | |

***Table S2:* Available options for each step in the radiomics model development pipeline**

| **Steps** |  | **Methods** |
| --- | --- | --- |
| Data balance |  | None |
|  |  | UpSampling |
|  |  | DownSampling |
| Normalization |  | None |
|  |  | Min-Max |
|  |  | Z-score |
|  |  | Mean |
| Dimension Reduction |  | Principle Component Analysis (PCA) |
|  |  | Pearson Correlation Coefficient (PCC) |
| Feature Selection |  | Analysis of Variance (ANOVA) |
|  |  | Recursive Feature Elimination (RFE) |
|  |  | Kruskal-Wallis Test |
| Classification |  | Support Vector Machine (SVM) |
|  |  | Least Absolute Shrinkage and Selection Operator (LASSO) |
|  |  | Linear Discriminant Analysis (LDA) |
|  |  | Random Forest (RF)  Decision Tree (DT) |
|  |  | Gradient Boosting |

***S2*:** **Details of the radiomics modeling pipelines**

*Data balance: Down sampling*

Down sampling can describe an entire process of bandwidth reduction (filtering) and sample-rate reduction. When the process is performed on a sequence of samples of a signal or other continuous function, it produces an approximation of the sequence that would have been obtained by sampling the signal at a lower rate (or density) (3).

*Normalization: Z-score*

The Z-score technique was used to obtain a standard normal distribution in the datasets. The mean value and the standard deviation were calculated. Each feature vector was subtracted from the mean value and divided by the standard deviation. After the Z-score process, each vector had a zero center and unit standard deviation.

*Dimension reduction: PCC*

To reduce the dimensions of the row space of the feature matrix, a Pearson correlation coefficient (PCC) was applied to each pair of two features. We compared the similarity of each feature pair. If the PCC value of the feature pair was larger than 0.99, we removed one feature randomly. After this process, the dimension of the feature space was reduced, and each feature was independent of the other features.

*Feature selection: ANOVA*

Analysis of variance (ANOVA) was used to select the features before building the model. ANOVA is a common method used to explore the significant features corresponding to the labels. The F-value was calculated to evaluate the relationship between the features and the label. We sorted the features according to the corresponding F-value and selected a specific number of features to build the model.

*Classifier: LASSO*

The Least Absolute Shrinkage and Selection Operator (LASSO) is a popular penalized regression method that may be used for variable selection of these high dimensional data.[^10^](https://www-ncbi-nlm-nih-gov-443.webvpn.bjmu.edu.cn/pmc/articles/PMC7449511/#R10) The LASSO method minimizes the residual sum of squares and places a bound on the sum of the absolute value of the coefficients (4). This bound is controlled by a shrinkage parameter that might cause some coefficients to be shrunk towards zero or set to be zero. The shrinking process might produce biased estimates, but it may improve both variable selection and interpretation. Nevertheless, when the covariates are subject to measurement error, variable selection by the LASSO method has been shown to be unstable (5).

**Reference**

1. Pyradiomics community. Customizing the extraction. 2016. Avaliable from: https://pyradiomics.readthedocs.io/en/latest/customization.html. [Accessed Jan 10,2020].

2. Pyradiomics community. Radiomics feature extraction in Python. 2016. Avaliable from: zhttps://github.com/radiomics/pyradiomics. [Accessed Oct 26,2020].

3. Zhu S, He Z, Meng X, Meng X, Zhou J, Guo Y, Zeng B. A New Polyphase Down-Sampling Based Multiple Description Image Coding. IEEE Trans Image Process. 2020 Apr 7.

4. Tibshirani R Regression shrinkage and selection via the Lasso. *J R Statist Soc B* 1996; 58: 267–288.

5.  Rosenbaum M and Tsybakov AB. Sparse recovery under matrix uncertainty. *Ann Stat* 2010; 38: 2620–2651.
